# Over-expression of glutamine synthase in focal nodular hyperplasia (part 1): Early stages in the formation support the hypothesis of a focal hyper-arterialisation with venous (portal and hepatic) and biliary damage

**DOI:** 10.1186/1476-5926-7-2

**Published:** 2008-02-29

**Authors:** Paulette Bioulac-Sage, Hervé Laumonier, Gaëlle Cubel, Jean Saric, Charles Balabaud

**Affiliations:** 1 Service d'Anatomie Pathologique Hôpital Pellegrin, CHU Bordeaux, France; 2 GREF Inserm U889, Université Bordeaux 2, France; 3 Service de Radiologie, Hôpital Saint André, CHU Bordeaux, France; 4 Pôle HGE, Hôpital Saint André, CHU Bordeaux, France

## Abstract

**Background:**

Most focal nodular hyperplasia (FNH) cases are diagnosed by chance. We studied a case of pre-FNH. We used glutamine synthase as an immunohistochemical marker for perivenous zones.

**Results:**

Neither fibrotic scars nor hepatocytic nodules surrounded by fibrosis with a ductular reaction were observed in the sections studied. Most sections generally displayed preserved architecture. The glutamine synthase-positive hepatocyte areas were wider than those observed in non-tumoural surrounding liver, and they tended to extend outwards. Portal tracts bordering the nodule were more fibrotic, with an absence of portal veins and ducts and with arterial proliferation often in proximity with large draining veins; isolated arteries were present and hepatic veins were rare in the nodule. These features appeared prior to the identification of other major criteria characteristics of FNH, thus supporting the "hypothesis of Wanless".

**Conclusion:**

The findings confirm that in FNH there is a portal tract injury leading to local portal vein injury. This leads to a cascade of events, including arterial venous shunts, ductular reaction, and scar formation.

## Background

The estimated prevalence of focal nodular hyperplasia(FNH) is 0.4 to 3% in a non-selected autopsy series and 0.3% in a clinical series. Most FNH cases are diagnosed by chance, but some are symptomatic. In contrast to hepatocellular adenomas, imaging techniques are sufficient for diagnosis in 70% of FNH cases. Histopathological examination is required for diagnosis in the few cases that have non-diagnostic imaging features [1,2]. In about two-thirds of cases, the FNH lesion is solitary. The typical histopathological features of classic FNH include a firm, well-delimited but not encapsulated lesion composed of hepatocellular nodules, a central scar, and radiating fibrous cords. The fibrous regions typically contain large dystrophic arteries and ductular reaction, usually associated with a lymphocytic infiltrate. FNH can be diagnosed from a liver biopsy. However, in some cases of FNH, the histopathological diagnosis may still be difficult, even in a resected specimen; this is because the definitive histopathological features may be absent, inconspicuous, or atypical. FNH can be associated with vascular abnormalities, including hepatic hemangiomas, which support vascular involvement in the pathogenesis of this lesion [3,4]. FNH has also been reported in patients with a variety of non-hepatic tumours and tumour-like conditions [4].

Recently, Wanless *et al*. hypothesized that the primary lesion of FNH is the result of a portal tract injury leading to injury of the local portal vein (PV) with secondary large arterial-PV shunts [5]. We studied the early stages of FNH formation to help validate this hypothesis. Thus, we reviewed a pre-FNH case previously published [6], taking advantage of glutamine synthase (GS) staining; this staining technique recognises hepatic veins surrounded by GS-positive hepatocytes or the distal part of zone 3 if the central vein is not visible [7].

## Case presentation

**Figure 1:**
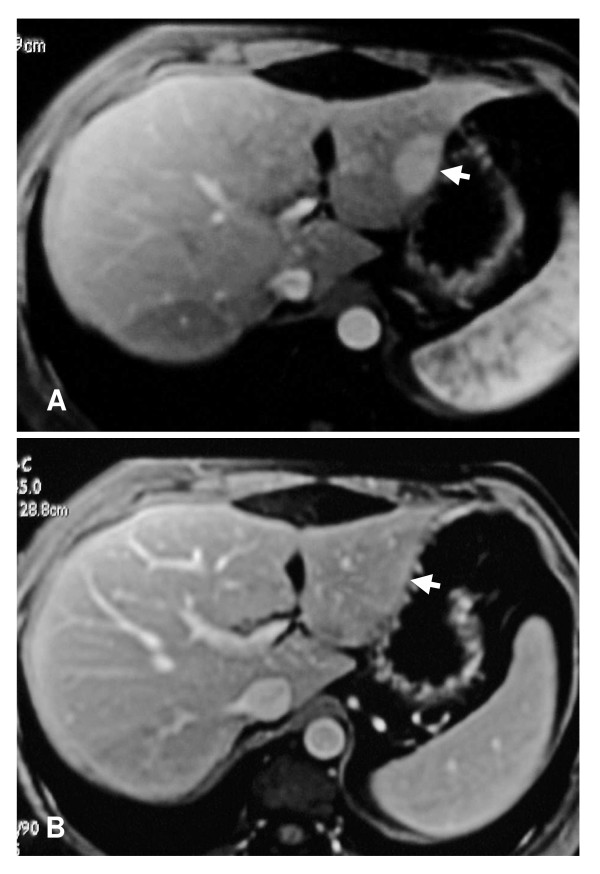
**Contrast-enhanced T1-weighted images**. They show strong enhancement of the lesion (white arrow) at arterial phase (A) and isointense signals relative to liver parenchyma at portal venous phase (B).

**Figure 2:**
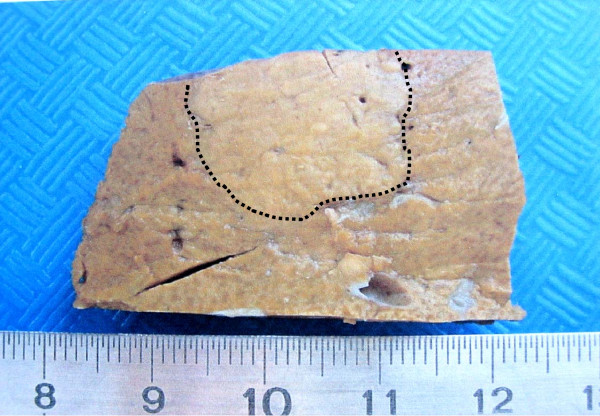
**Macroscopic view (A, B) of the nodule (formalin fixed tissue)**. Then nodule appears slightly clearer than the surrounding liver parenchyma (dotted line).

A white female patient born in 1950 was reported to have vesicular polyps for several years. In 2000, she presented with biliary colic, and a stone was detected in her gallbladder. A hypoechoic nodule was also identified in the left lobe (Fig. 1). Isointense patterns relative to liver parenchyma on T1-weighted and T2-weighted images with strong enhancement at arterial phase, and no wash-out at portal venous phase favoured a benign liver nodule. However, the diagnosis of FNH could not be achieved with certainty, due to the absence of all the imaging criteria and, above all, the absence of a central scar. The diagnosis of adenoma could not be excluded even if homogeneous and isointense patterns on both T1 and T2 weighted images are considered rare for this kind of lesion. Furthermore, there was no evidence of fat deposits within this lesion (Fig. 1). The patient had been taking oral contraceptives for 10 years. Liver function tests for the patient were normal. A left hepatectomy and a cholecystectomy were performed at the same time. The resected nodule was barely visible on an examination of gross anatomy, and was slightly clearer than the surrounding liver parenchyma (Fig. 2). This nodule was the base of this study.

We sampled three representative sections of the nodule whole surface and non-tumoural surrounding tissue. The following stains and immunostains were performed on serial sections from 2 blocks: H&E, Masson's trichrome, cytokeratines (CK) 7 and 19, α-smooth muscle actin (SMA), and GS. Additional immunostaining included CD34 for characterising capillarised sinusoids, CRBP1 (cellular retinol binding protein-1) for hepatic stellate cells, CD3 and CD20 for T- and B-lymphocytes, respectively, and CD68 for histiocytes.

**Figure 3:**
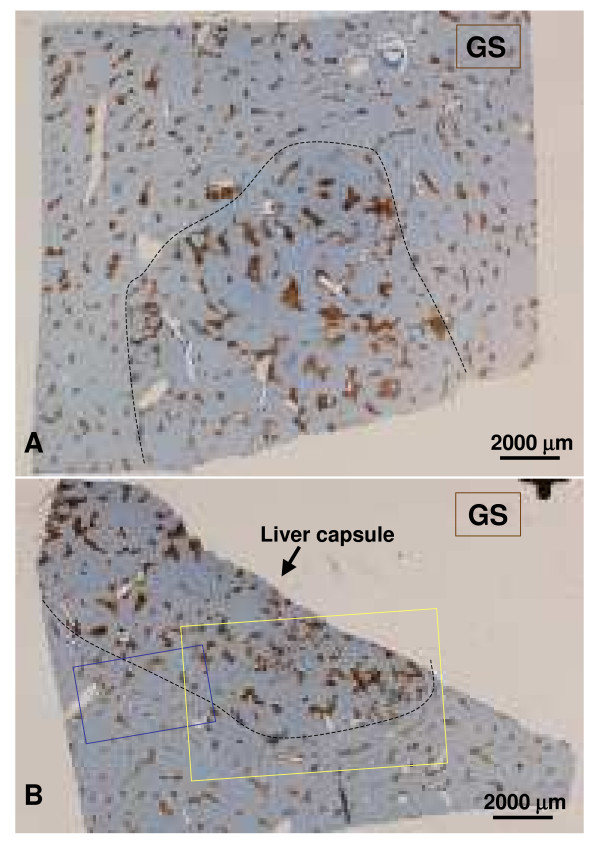
**Two sections (A, B) of the nodule**. The GS staining allows the distinction between non-nodular tissue and the nodule. The broken line delineates the frontier. Figures 9, 20–25 are from section A. The upper figure is from non-nodular tissue and the lower figure is from the nodular area. Figures 4-6 are from section B (blue rectangle). Figures 7, 8, 10-19 are from the same section (yellow rectangle). The upper and lower microphotographs illustrate the same area (serial sections).

The non-tumoural liver was steatotic (30%), and the nodule was less steatotic in comparison. Early stage FNH formation was diagnosed due to frank ductular reaction, observed around the arteries in two small areas (see Fig. 3, [6]). We observed no fibrotic scars, or hepatocytic nodules surrounded by fibrosis with ductular reaction in the sections studied.

**Figure 4:**
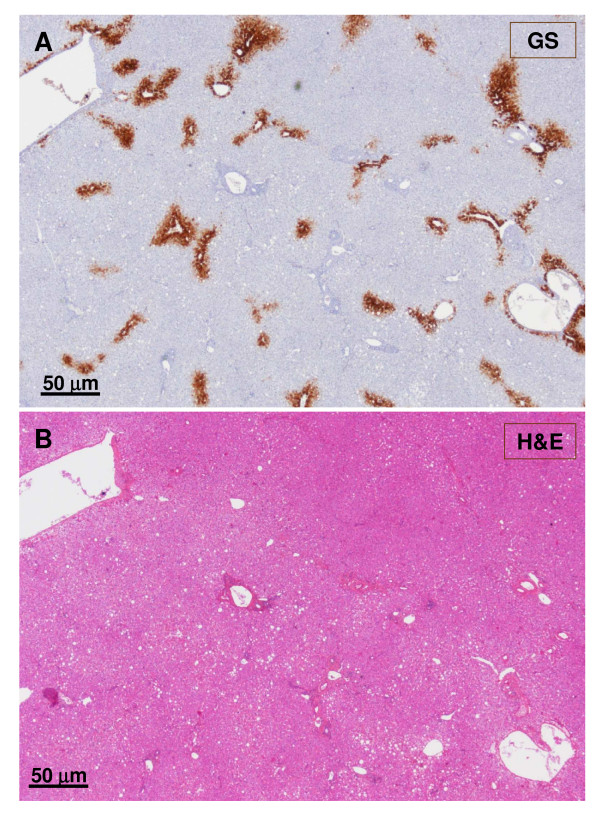
**Junction between non-nodular tissue (lower part) and nodule (upper part)**. The upper micrograph (A) displays GS staining, and the lower micrograph (B) shows H&E staining. The GS stained area is wider in the nodular part than in the non-nodular part. The lobular structure of the liver is not easily visible in the nodular part.

**Figure 5:**
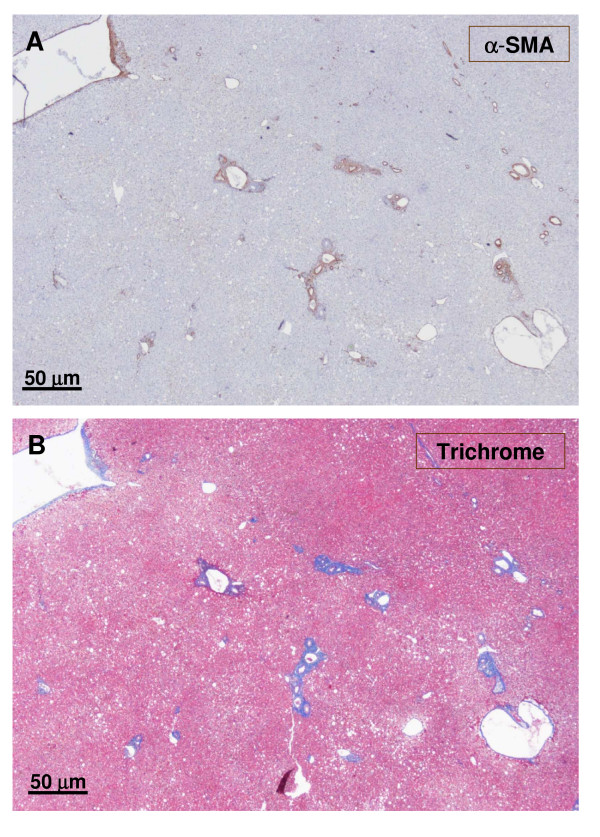
**Junction between non-nodular tissue (lower part) and nodule (upper part)**. The upper micrograph (A) shows α-SMA staining, and the lower micrograph (B) exhibits trichrome staining. At the junction between the nodular and non-nodular part, portal tracts are more fibrotic (trichrome). The nodular parts contain many isolated arteries.

**Figure 6:**
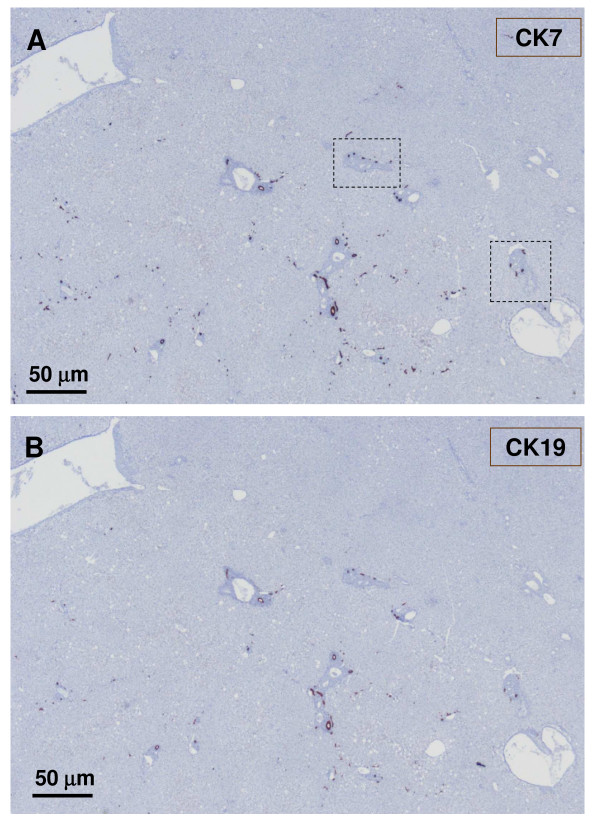
**Junction between non-nodular tissue (lower part) and nodule (upper part)**. The upper micrograph (A) shows CK7 staining, and the lower micrograph (B) displays CK19 staining. There is no (CK19) or less (CK7) biliary cells in the nodular part compared to the non-nodular part.

**Figure 7:**
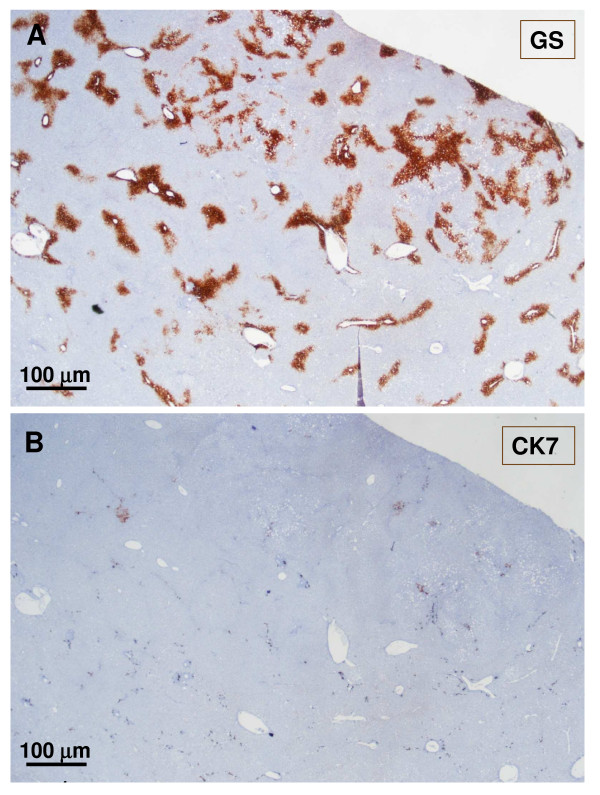
**Another area of the previously described upper section**. The figure shows the upper section as previously described (Fig. 4-5), but in another area. The upper micrograph (A) shows GS staining, and the lower micrograph (B) has CK7 staining. It confirms the extension of GS staining and the decrease number of biliary cells in the nodule.

**Figure 8:**
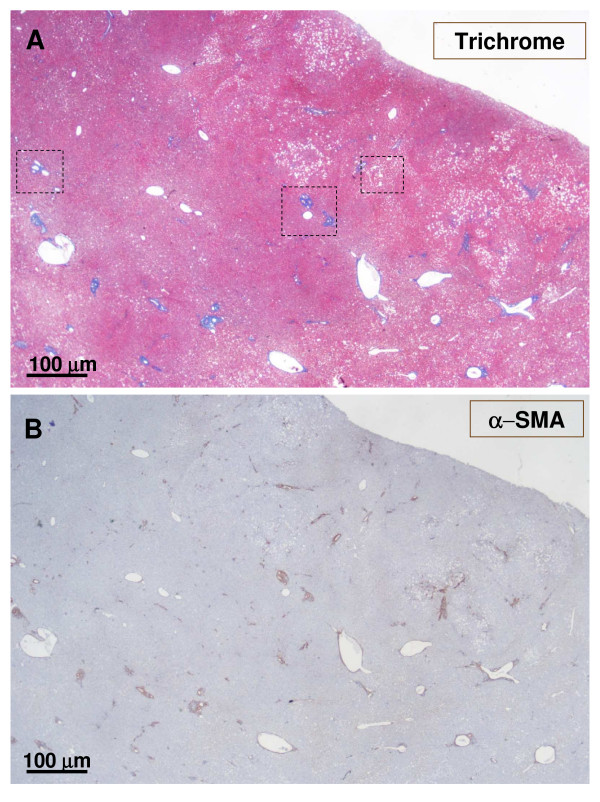
**Another area of the previously described upper section**. The figure shows the upper section as previously described (Fig. 4-5), but in another area. The upper micrograph (A) was trichrome stained, and the lower micrograph (B) was α-SMA stained. It confirms the proliferation of arteries in the nodules and the presence of fibrotic portal tracts at the periphery of the nodule.

**Figure 9:**
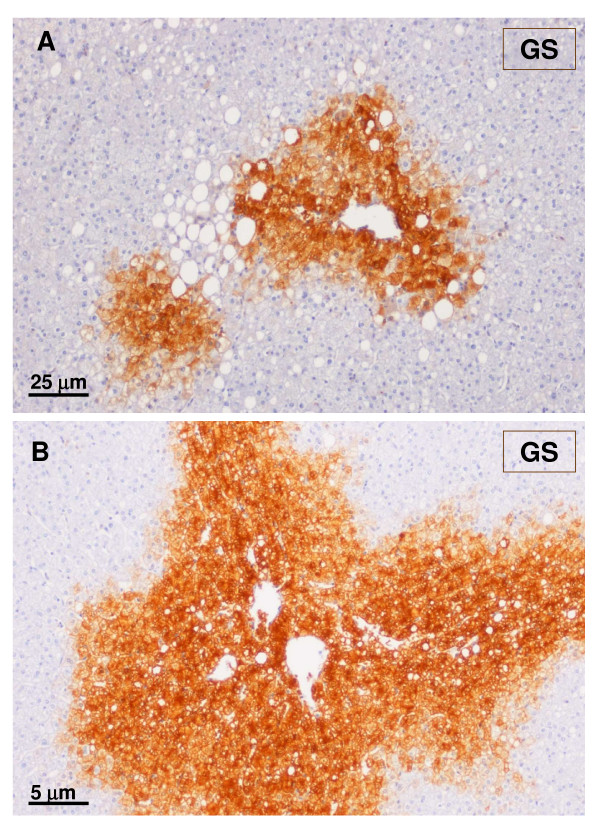
**Glutamine synthase immunostaining**. A: Normal expression of GS around the central vein (non-nodular tissue). B: Extended staining around hepatic veins in the nodule.

**Figure 10:**
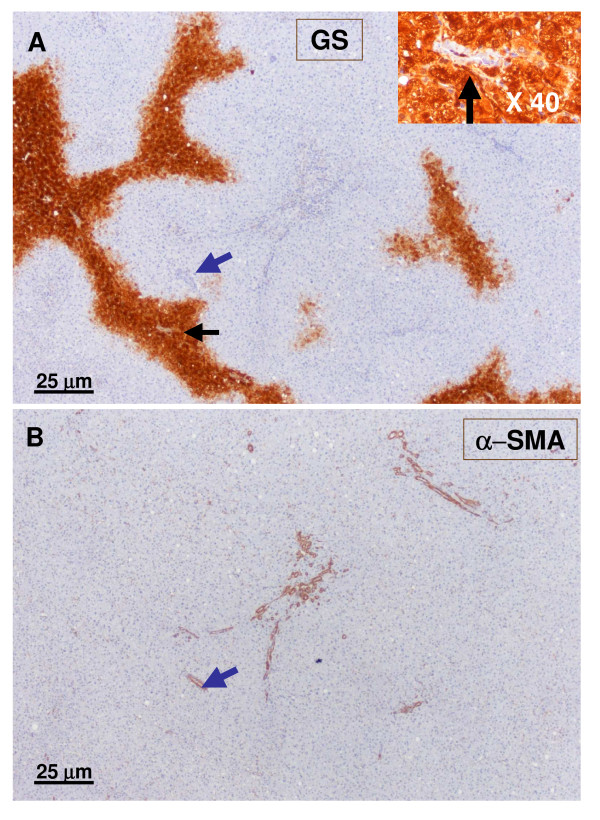
**Typical GS staining in the nodule**. A – Higher magnification. B – Lower magnification. The nodule partly surrounds arterial vessels – these were better seen using α-SMA staining. The blue arrow indicates an artery, whereas the black arrow indicates a small hepatic vein (insert) with a thick wall and a narrow almost absent lumen.

**Figure 11:**
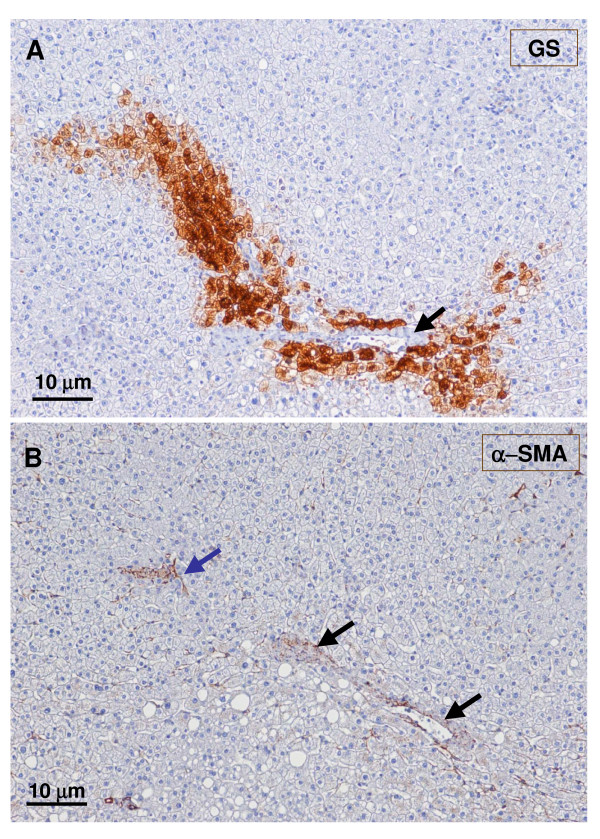
**Small hepatic veins**. A – GS staining. B – α-SMA staining. In the rather faint GS-positive area, a small hepatic vein (black arrow) was identified on the right. The vascular lumen was no longer observed on the left (blue arrow). No artery was seen in the vicinity.

**Figure 12:**
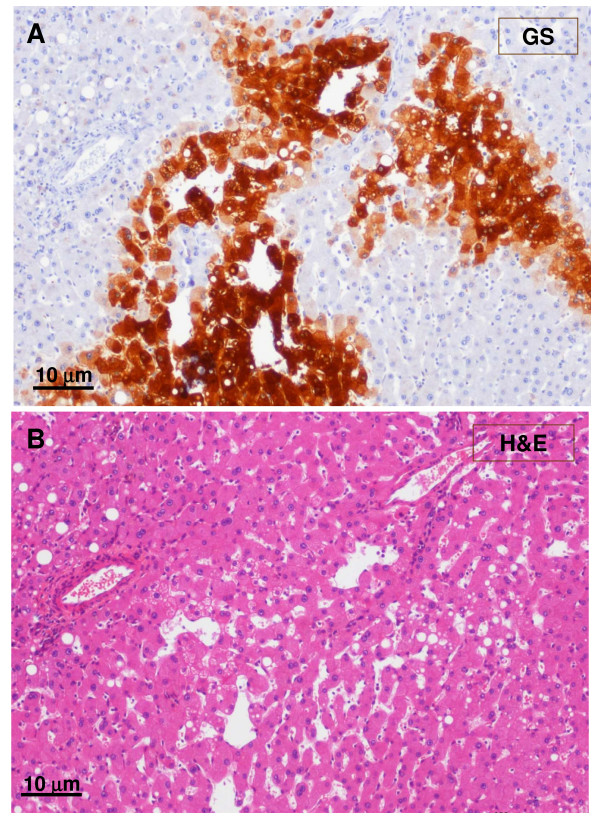
**GS-positive hepatocytes**. A – GS staining. B – H&E staining. A clump of GS-positive hepatocytes (see boxes on Fig. 8) was observed around dilated sinusoids. Hepatic veins were not visible in this area.

**Figure 13:**
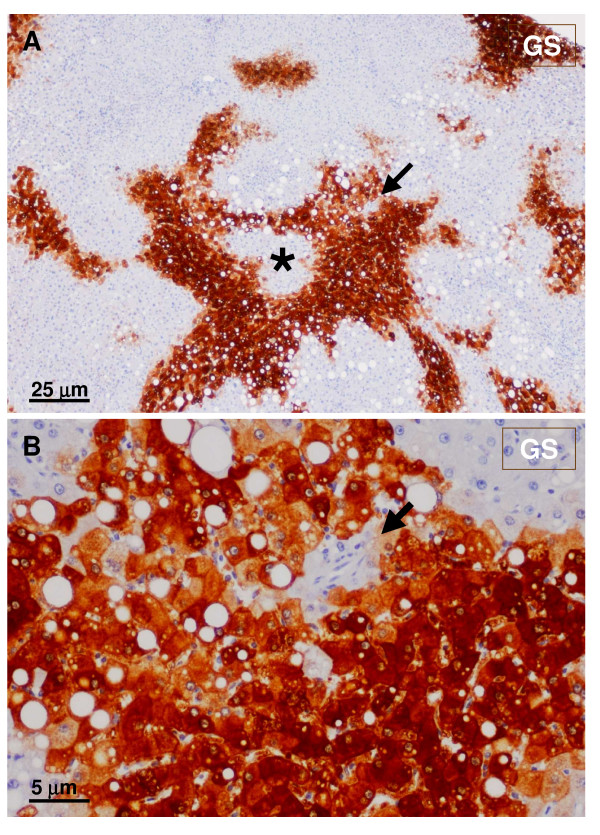
**GS-positive staining**. A – Lower magnification. B – Higher magnification The GS staining surrounds unstained hepatocytes (asterisk). Hepatic veins were not visible; a small artery (arrow) was seen at the edge of the GS-positive area.

**Figure 14:**
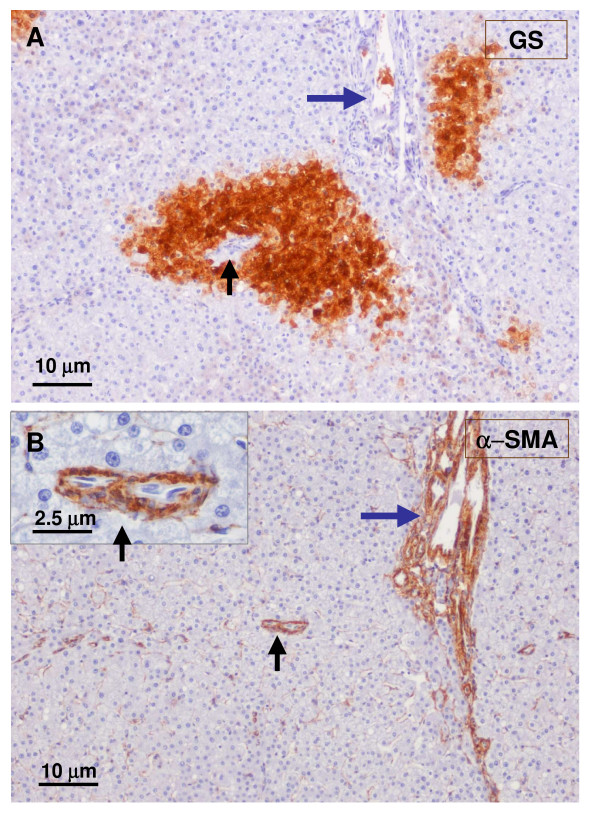
**GS positive foci**. GS foci (A – upper panel) contained a small artery (black arrow and insert) very close to arterial branches (blue arrows), better seen in a α-SMA staining (B – lower panel).

**Figure 15:**
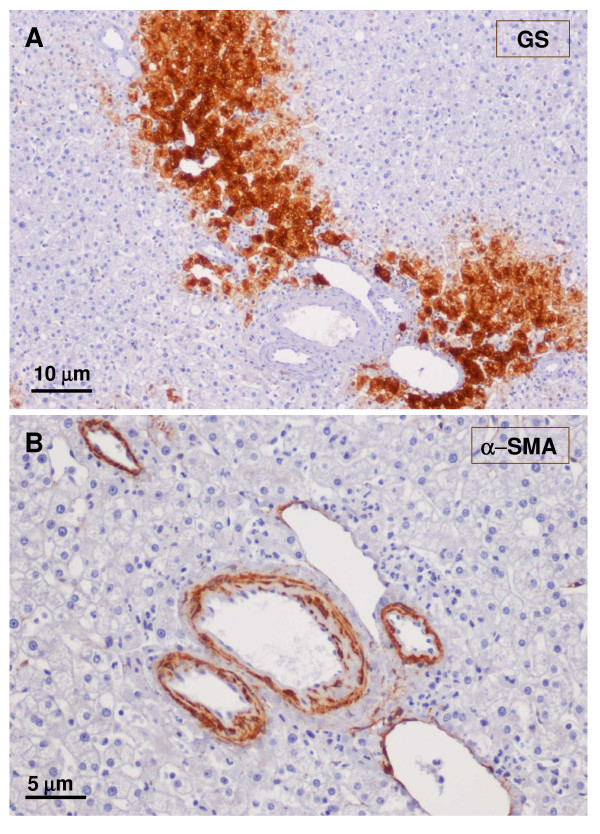
**GS and α-SMA staining**. A closer view (see Fig. 8, box on the left): upper micrograph (A) shows GS staining, and lower micrograph (B) shows α-SMA staining. Arteries (α-SMA staining) are close to large hepatic veins (GS staining).

**Figure 16:**
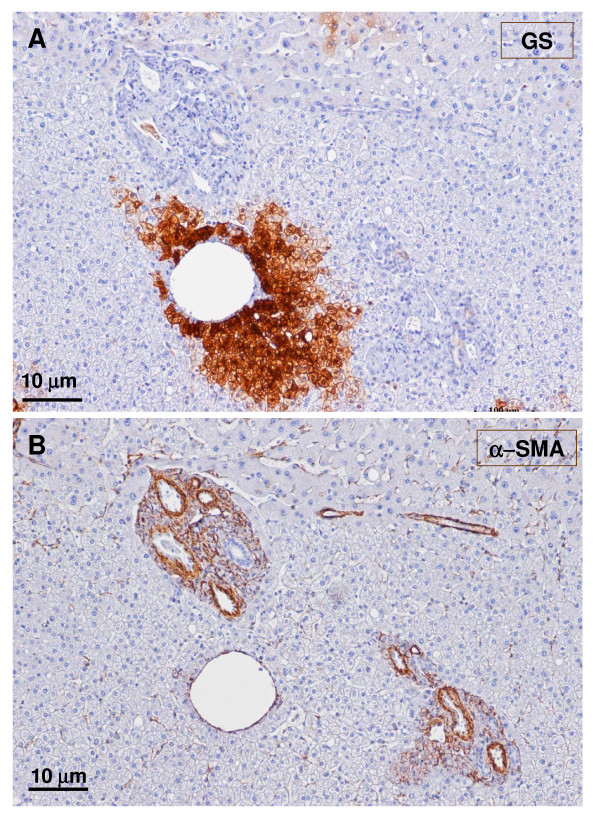
**GS and α SMA staining**. A closer view (see Fig. 8 box in the middle): upper micrograph (A) has GS staining, and lower micrograph (B) displays α-SMA staining. It illustrates another example of the close vicinity between arteries (α-SMA staining) and hepatic veins (GS staining).

**Figure 17:**
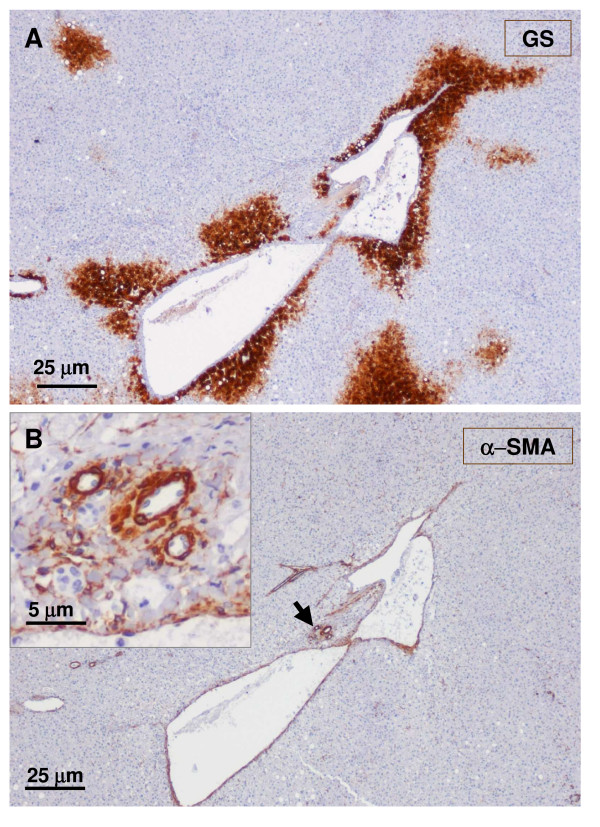
**Arterial vessels**. A – GS staining. B – α-SMA. Arterial vessels (arrow, insert) were in close contact with the large hepatic vein partly surrounded by GS-positive hepatocytes.

**Figure 18:**
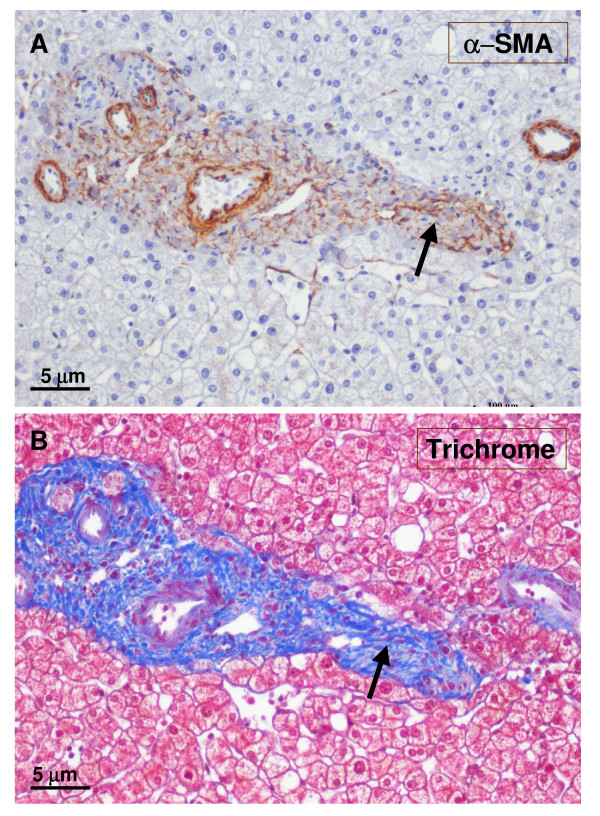
**Fibrotic portal tract**. A fibrotic portal tract (see Fig. 6, box on the left) is observed at higher magnification. There are several arteries and a few ducts and ductules (A – α-SMA staining). A possible portal vein remnant can also be identified (B – trichrome staining) (arrow).

**Figure 19:**
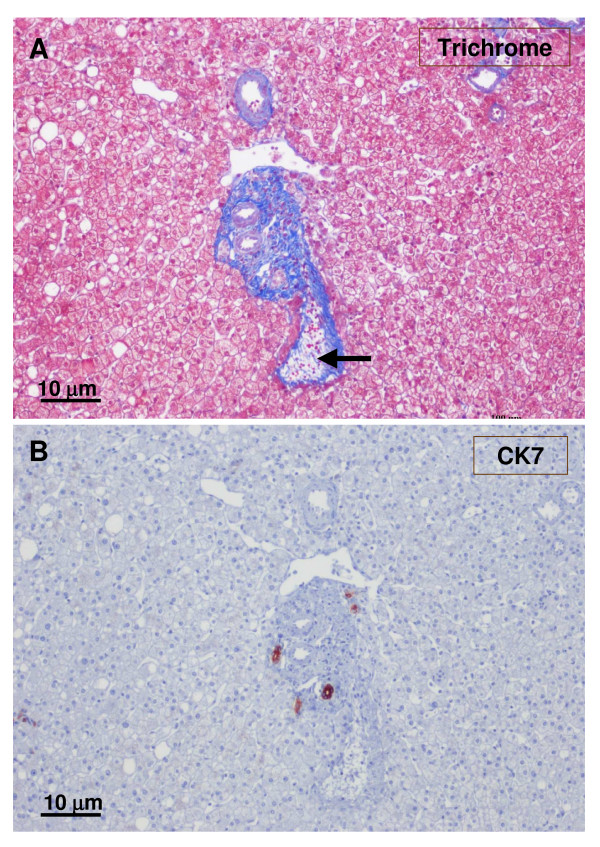
**Fibrotic portal tract**. A fibrotic portal tract (see Fig. 6, box on the right) is observed at higher magnification. The wall of the portal vein appears damaged (A – trichrome staining). Remnants of small bile ducts can also be identified (B – CK7 staining), and an aberrant vessel is at the top of the figure.

**Figure 20:**
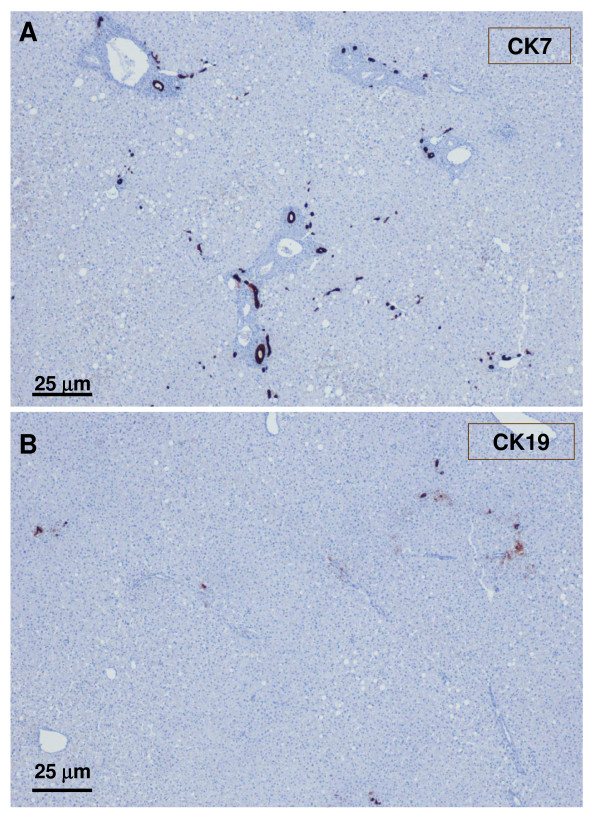
**Non-tumoral liver and nodule**. A – non-tumoral liver, and B – nodule. Ducts and ductules (CK7 and CK19) were more difficult to identify and less numerous in the tumoral liver than in the non-tumoral liver.

Most sections generally displayed preserved architecture (Fig. 3, 4, 5, 6, 7 and 8). Distinctions between nodular and non-nodular tissues were made using GS immunostaining (Fig. 3, 4). Inside the nodule, GS-positive hepatocyte areas were wider around central veins if visible (Fig. 9), and tended to extend outwards (Fig. 3, 4, 7, 10). Surprisingly, hepatic veins (HV) in some GS-positive areas were missing or had undergone fibrosis (Fig. 10, 11). GS staining was sometimes also present around thin vessels, resembling dilated sinusoids (Fig. 12). Also in some GS-positive areas, only arteries were visible (Fig. 13, 14). GS staining was sometimes also present around thin vessels, resembling dilated sinusoids (Fig. 12). However, isolated arteries either along septa or in the parenchyma, often in close proximity (Fig. 15, 16) or in contact with large draining vessels, were visible (Fig. 17). At the nodule periphery, portal tracts were slightly fibrous, containing thicker arteries and remnants of PV, and fewer ducts and ductules (Fig. 18, 19). No true portal tracts, and no bile ducts or ductules were observed in the nodule (Fig. 20).

**Figure 21:**
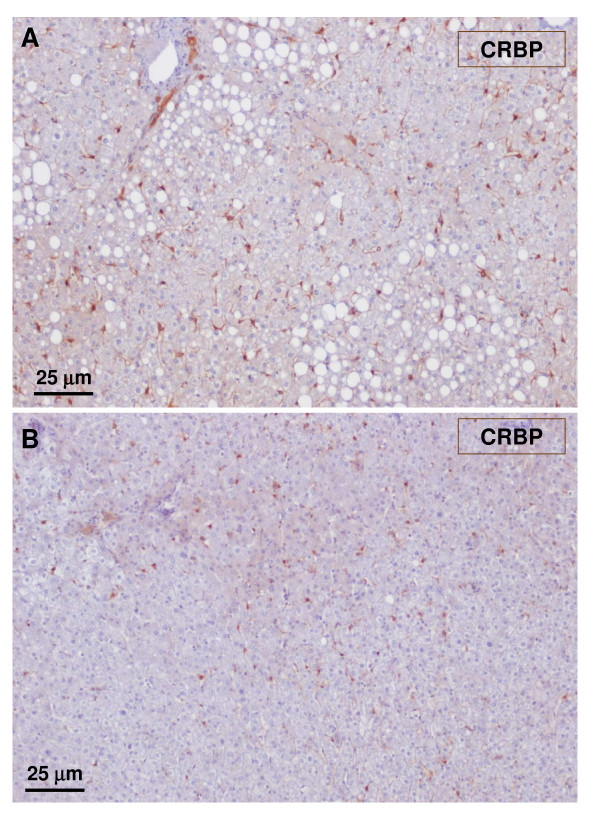
**Non-tumoral liver and nodule**. A – non-tumoral liver, and B – nodule. Hepatic stellate cells identified by CRBP-1 are less visible in the tumoral liver than in the non-tumoral liver.

**Figure 22:**
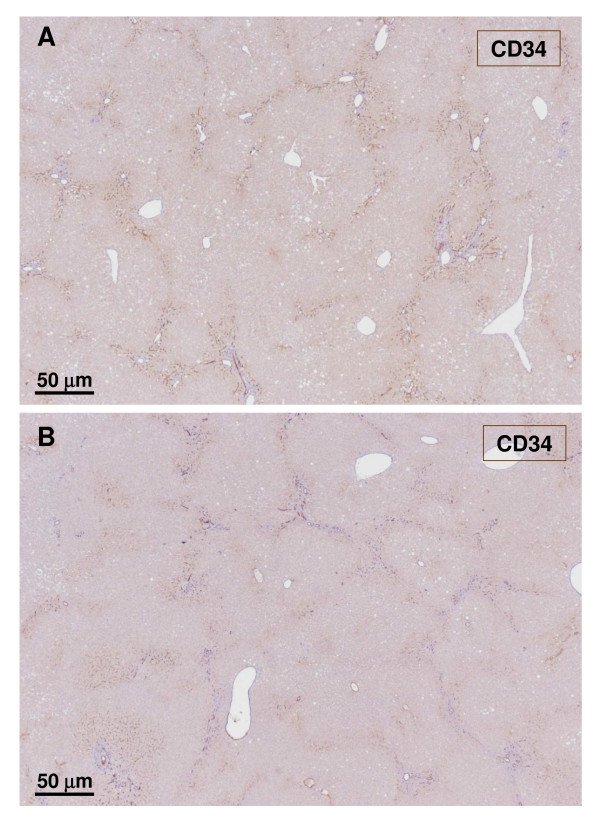
**Non-tumoral liver and nodule**. A – non-tumoral liver, and B – nodule. There is no major difference regarding capillarisation of sinusoids in periportal and septal areas between the tumoral liver and the non-tumoral liver.

**Figure 23:**
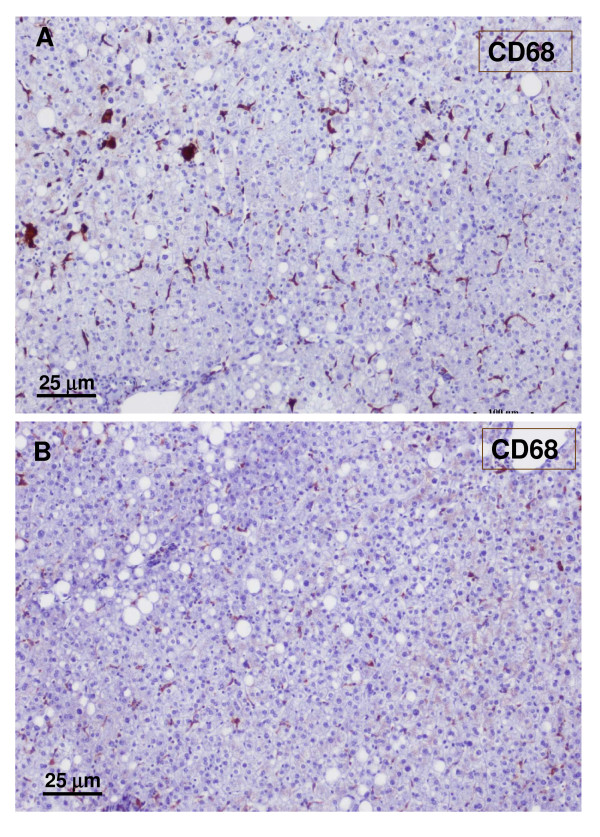
**Non-tumoral liver and nodule**. A – non-tumoral liver, and B – nodule. There are less macrophages (CD68) in the tumoral liver than in the non-tumoral liver.

**Figure 24:**
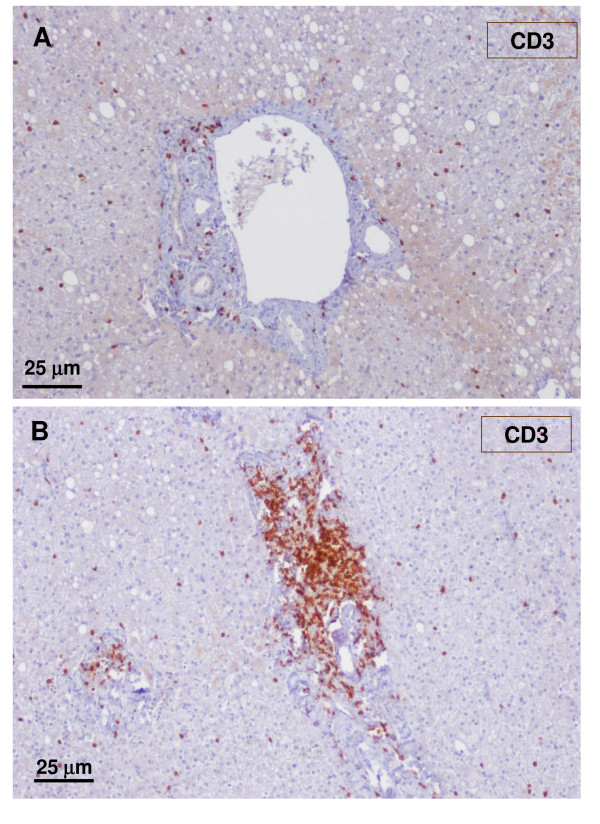
**Non-tumoral liver and nodule**. A – non-tumoral liver, and B – nodule. There are more CD3 lymphocytes in the tumoral liver than in the non-tumoral liver.

**Figure 25:**
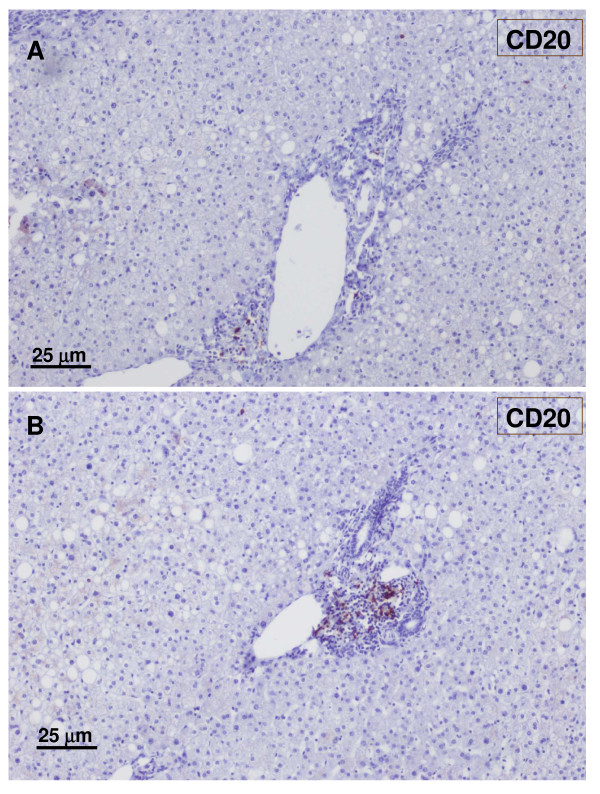
**Non-tumoral liver and nodule**. A – non-tumoral liver, and B – nodule. There are more CD20 lymphocytes in the tumoral liver than in the non-tumoral liver.

The nodule had fewer hepatic stellate cells than those observed in non-tumour parenchyma, (Fig. 21); these stellate cells were not activated (Fig. 15, 16, 17 and 18), i.e., they were α-SMA negative. There was no major difference regarding capillarisation of sinusoids in periportal and septal areas, between the tumoral liver and the non-tumoral (Fig. 22). Portal tracts of the nodule also had fewer macrophages (Fig. 23) and more B and T lymphocytes (Fig. 24, 25) than those of non-tumour parenchyma.

## Discussion

FNH is frequently observed in tertiary referral centres. The diagnosis is usually easy using imaging techniques, such as ultrasound contrast agents, or MRI. If imaging techniques or biopsies cannot exclude adenoma [1], a diagnosis can also be made using the resected specimen. We found the use of various immunohistochemical methods, including LFABP (cellular retinol binding protein), β-catenin, SAA (serum amyloid A) and GS [8], very useful for differentiating between FHN and adenoma in difficult cases.

Glutamine synthase, physiologically expressed in one or two hepatocyte plates located immediately beside the central veins in normal liver, is over-expressed in FNH with over-stained hepatocytes generally forming large areas; often anastomosed in a "map-like" pattern occasionally centred by central veins, whereas GS positive areas remain distant from fibrous bands. This zonal pattern of GS expression in FNH strongly differs from the homogeneous staining usually observed in adenomas and adenomas in which β-catenin is mutated [8].

Surprisingly, there is not much data in relation to FNH formation [6]. In this case, the lobular structure was generally preserved in each of the sections studied, and we observed no ductular reaction and no fibrosis. Hepatic veins were identified from portal tracts with certainty, as a result of GS localization, and isolated arteries were visually detected well with α-SMA, thus providing a better understanding of FNH.

From this study, it is clear that in early stages of FNH formation there are various features: a) at the border, there were abnormal portal tracts, which were more fibrotic, and there was also an absence of portal veins and ducts and arterial proliferation, often in proximity with large draining veins; b) isolated arteries in the lesion; c) an absence or rarefaction of hepatic veins. All these features appeared prior the identification of other major characteristics of FNH, i.e., nodules circled by fibrous bands, ductular reaction, abnormal vessels and several arteries in fibrotic bands.

These observations support suggestions by Wanless et al. [5] and confirm that in FNH there is a portal tract injury leading to local PV injury. Wanless' group has suggested that the PV injury (from thrombosis or phlebitis) leads to a cascade of events, such as secondary large arterio-PV shunts. Arterio-PV shunts lead to the arterialisation of PV with irregular intimal thickening resembling a dystrophic artery. Wanless and colleagues have also suggested that duct loss is related to portal tract changes, and that the entry of enlarged arteries in the hepatic venous bed could be the consequence of congestive injury.

It is not possible to fully understand the pathogenesis of FNH from a single case. It is possible, however, from our current understanding of the role of the hepatic artery in the normal liver [9] to propose a direct role of this vessel in FNH formation.

In normal livers, the artery within the portal tract supplies three compartments: the peribiliary vascular plexus, the portal tract interstitium, and the portal vein wall. However, the artery outside the portal tract (termed isolated artery) supplies two compartments: the hepatic capsule and the hepatic vein wall [9].

The prime cause of FNH may be arterial, due to the lack of branches to various tributaries (portal, hepatic veins and ducts). This lack of branches may lead to conditions that are either primary (malformative focal arterial disease) or secondary to inflammation (as suggested by an increased number of inflammatory cells in portal tracts); this in turn leads to the disappearance of portal vein, bile ducts, and hepatic veins, subsequently resulting in the enlargement and proliferation of arteries, in venous shunts (portal and hepatic) and in all other features described by Wanless et al. [5]. Disappearance of bile ducts may lead to cholestasis, and therefore to ductular reaction and fibrosis.

The aim of this study was not to confirm the presence of arterio-venous shunts (portal vein or hepatic vein). The existence of these shunts proposed by Wanless' group is however very likely (proximity of artery to hepatic vein, and dystrophic arterial vessels in portal tracts).

The suggestion of an arterial malformation is consistent with the possibility of discovering FNH during foetal life and in infancy, the stability of the lesions over time, the association with other vascular malformations, and the singular or multiple nature of nodules [1].

## Conclusion

Our observations confirm that in FNH there is a portal tract injury defined by the disappearance of portal vein, bile ducts, and hepatic veins, subsequently leading to the enlargement and proliferation of arteries, and to venous shunts (portal and hepatic). The prime cause of FNH may be arterial.

## Competing interests

The author(s) declare that they have no competing interests.

## Authors' contributions

PB-S and CB designed the study and wrote the paper. HL performed the imaging technique. GC performed the immunohistochemistry. JS was in charge of the patient and performed the surgery. All authors read and approved the final manuscript.

## Consent

Informed consent was obtained from the patient for publication of this case report.
